# Ovarian Sertoli-Leydig Cell Tumors: Epidemiological, Clinical and Prognostic Factors

**DOI:** 10.1055/s-0039-1693056

**Published:** 2019-07-10

**Authors:** Beatriz Guerreiro Ruiz Castro, Cristiano de Pádua Souza, Carlos Eduardo Mattos da Cunha Andrade, Marcelo de Andrade Vieira, Diocésio Alves Pinto de Andrade, Ricardo dos Reis

**Affiliations:** 1 Faculdade de Ciências da Saúde de Barretos Dr. Paulo Prata, Barretos, SP, Brazil; 2 Gynecologic Clinical Oncology Department, Hospital do Câncer de Barretos, Barretos, SP, Brazil; 3 Gynecologic Oncology Department, Hospital do Câncer de Barretos, Barretos, SP, Brazil; 4 InORP ONCOCLÍNICAS Group (Instituto Oncológico de Ribeirão Preto), Ribeirão Preto, SP, Brazil

**Keywords:** ovarian neoplasms, Sertoli-Leydig cell tumor, oncologic prognosis, neoplasias ovarianas, tumor de células de Sertoli-Leydig, prognóstico oncológico

## Abstract

**Objective** To describe a series of cases of ovarian Sertoli-Leydig cell tumors (SLCTs).

**Methods** Retrospective review of 12 cases of SLCT treated at the Hospital do Câncer de Barretos, Barretos, state of São Paulo, Brazil, between October 2009 and August 2017.

**Results** The median age of the patients was 31 years old (15–71 years old). A total of 9 patients (75.0%) presented symptoms: 8 (66.7%) presented with abdominal pain, 5 (41.7%) presented with abdominal enlargement, 2 (16.7%) presented with virilizing signs, 2 (16.7%) presented with abnormal uterine bleeding, 1 (8.3%) presented with dyspareunia, and 1 (8.3%) presented with weight loss. The median preoperative lactate dehydrogenase (LDH) was 504.5 U/L (138–569 U/L), alpha-fetoprotein (AFP) was 2.0 ng/ml (1.1–11.3 ng/ml), human chorionic gonadotropin (β-hCG) was 0.6 mUI/ml (0.0–2.3 mUI/ml), carcinoembryonic antigen (CEA) was 0.9 ng/ml (0.7–3.4 ng/ml), and cancer antigen 125 (CA-125) was 26.0 U/ml (19.1–147.0 U/ml). All of the tumors were unilateral and surgically treated. Lymphadenectomy was performed in 3 (25.0%) patients, but none of the three patients submitted to lymphadenectomy presented lymph node involvement. In the anatomopathological exam, 1 (8.3%) tumor was well-differentiated, 8 (66.7%) were moderately differentiated, and 3 (25.0%) were poorly differentiated. A total of 5 (55.6%) tumors were solid-cystic, 2 (22.2%) were purely cystic, 1 (11.1%) was cystic with vegetations, and 1 (11.1%) was purely solid, but for 3 patients this information was not available. The median lesion size was 14.2 cm (3.2–23.5 cm). All of the tumors were at stage IA of the 2014 classification of the International Federation of Gynecology and Obstetrics (FIGO). A total of 2 (16.7%) patients received adjuvant treatment; 1 of them underwent 3 cycles of paclitaxel and carboplatin every 21 days, and the other underwent 4 cycles of ifosfamide, cisplatin and etoposide every 21 days. None of all of the patients had recurrence, and one death related to complications after surgical staging occurred.

**Conclusion** Abdominal pain was the most frequent presentation. There was no ultrasonographic pattern. All of the SLCTs were at stage IA, and most of them were moderately differentiated. Relapses did not occur, but one death related to the surgical staging occurred.

## Abstract (pt)

**Resumo:**

**Objetivo** Descrever uma série de casos de tumores de células de Sertoli-Leydig (TCSLs) ovarianos.

**Métodos** Revisão retrospectiva de 12 casos de TCSL tratados no Hospital de Câncer de Barretos entre outubro de 2009 e agosto de 2017.

**Resultados** A mediana de idade foi 31 anos (15–71 anos). Um total de 9 pacientes (75,0%) apresentaram sintomas: 8 (66,7%) apresentaram dor abdominal, 5 (41,7%) apresentaram aumento abdominal, 2 (16,7%) apresentaram virilização, 2 (16,7%) apresentaram sangramento uterino anormal, 1 (8,3%) apresentou dispareunia, e 1 (8,3%) apresentou emagrecimento. A mediana de desidrogenase láctica (DHL) foi 504,5 U/L (138–569 U/L), alfafetoproteína (AFP) foi 2,0 ng/ml (1,1–11,3 ng/ml), gonadotrofina coriônica humana (β-hCG) foi 0,6 mUI/ml (0,0–2,3 mUI/ml), antígeno carcinoembrionário (CEA) foi 0,9 ng/ml (0,7–3,4) ng/ml, e antígeno cancerígeno 125 (CA-125) foi 26,0 U/ml (19,1–147,0 U/ml), todos pré-operatórios. Todos os tumores foram unilaterais e tratados cirurgicamente. Realizou-se linfadenectomia em 3 (25,0%) pacientes, porém, nenhuma das três apresentou acometimento linfonodal. No exame anatomopatológico, 1 tumor (8,3%) era bem diferenciado, 8 (66,7%) eram moderadamente diferenciados, e 3 (25,0%) eram pouco diferenciados. Um total de 5 (55,6%) tumores eram sólido-císticos, 2 (22,2%) eram puramente císticos, 1 (11,1%) era cístico com vegetações, e 1 (11,1%) era puramente sólido, mas para 3 pacientes estas informações não estavam disponíveis. A mediana da dimensão da lesão foi 14,2 cm (3,2–23,5 cm). Todos os tumores eram estádio IA de acordo com a classificação de 2014 da Federação Internacional de Ginecologia e Obstetrícia (FIGO, na sigla em inglês). Duas (16,7%) pacientes receberam adjuvância; uma realizou 3 ciclos de paclitaxel e carboplatina a cada 21 dias, e a outra 4 ciclos de ifosfamida, cisplatina e etoposide a cada 21 dias. Dentre todas as pacientes, nenhuma apresentou recidiva e houve um óbito relacionado a complicações após estadiamento cirúrgico.

**Conclusão** Dor abdominal foi a apresentação mais frequente. Todos os TCSLs eram estádio IA e a maioria era moderadamente diferenciada. Não ocorreram recidivas, mas ocorreu um óbito relacionado ao estadiamento cirúrgico.

## Introduction

Ovarian Sertoli-Leydig cell tumors (SLCTs) are part of the sexual cord neoplasms and represent < 0.5% of all ovarian tumors.^1^ This type of tumor predominates in the 2^nd^ and 3^rd^ decades of life and usually presents with hormonal changes, including signs of virilization, such as amenorrhea, hirsutism, acne, and male pattern of pilification.^2^ ^3^ ^4^ ^5^ ^6^ ^7^ ^8^ These characteristics are due to an increase in androgen production by tumor cells. In rare cases, signs of hyperestrogenism may also occur, such as postmenopausal bleeding and endometrial hyperplasia.^8^ ^9^ This increase in estrogen levels may be due to secretion of the hormone by tumor cells or to the peripheral conversion of testosterone produced by the tumor to estrogen through the action of aromatase. In patients without hormonal manifestations, the typical presentation of the disease consists of abdominal pain and increased abdominal circumference, usually with a palpable adnexal mass at physical examination.^10^ Some authors suggest that the preoperative diagnosis in patients without association of signs of virilization and palpable abdominal mass would be practically impossible.^2^

Microscopically, SLCTs can be divided into well-, moderately, and poorly differentiated, with or without heterologous elements and/or retiform pattern.^3^ ^11^ Along with staging, the degree of histological differentiation represents some of the prognostic factors described in the literature.^12^ The malignant potential in well-differentiated tumors is practically null and increases substantially in those with lower degrees of differentiation.^3^ ^13^ Bhat et al^6^ reported a relationship between staging and degree of histological differentiation. In the present study, 85.7% and 75.0%, respectively, of the well- and moderately differentiated tumors corresponded to stage IA, whereas, of the poorly differentiated, > 50.0% of the cases were stage IC or more advanced.^6^

Most of the ovarian SLCTs are unilateral, and their treatment is preferably surgical.^3^ ^8^ ^14^ ^15^ Unilateral salpingo-oophorectomy is the procedure of choice for patients that want to preserve their fertility.^5^ ^7^ ^8^ ^11^ For patients without fertility preservation, as well as in advanced stages, total hysterectomy and bilateral salpingo-oophorectomy (BSO) or cytoreductive surgery should be recommended.^4^ ^7^ As lymph node metastases are rare in ovarian SLCTs, lymphadenectomy can be omitted as part of the surgical staging in these patients.^16^ ^17^ In addition, relapse is uncommon in SLCTs, and adjuvant chemotherapy should be reserved for patients with risk factors such as moderately or poorly differentiated tumors, patients with advanced stages, or in recurrence.^4^ ^14^

The low incidence of this type of ovarian neoplasia explains the paucity of data about the clinical behavior of SLCTs and their oncology outcomes. Thus, the purpose of the present study is to describe a series of ovarian SLCT cases managed in a tertiary cancer center.

## Methods

### Research Design

After approval from the institutional review board of the Pio XII Foundation – Hospital do Câncer de Barretos (HCB) number 1511/2017, an observational retrospective study was conducted based on the analysis of a convenience sample of all of the patients diagnosed with ovarian SLCTs treated at the Department of Gynecology Oncology of the HCB between October 2009 and August 2017.

### Population

All of the patients with a diagnosis of ovarian SLCT treated at the Department of Gynecology Oncology of the HCB between October 2009 and August 2017 were included. There were no exclusion criteria, because we included a series of cases within the time frame.

### Research Variables

In the present study, we evaluated epidemiological, clinical, and prognostic data related to ovarian SLCTs. The data were collected through the review of the medical records of patients with ovarian SLCTs, including age, date of birth, signs and symptoms, comorbidities, hormonal profile, serum tumor markers, imaging examination, type of surgery performed, degree of histological differentiation, staging, adjuvant treatment, length of follow-up, and occurrence of relapses or deaths. Tumor staging was based on the International Federation of Gynecology and Obstetrics (FIGO) classification of 2014.^18^ The length of follow-up was calculated from the date of the first surgery until the date of the last medical record information.

### Statistical Analysis

Descriptive statistics were used to characterize the sample. Initially, quantitative variables were described by mean and standard deviation (SD) or median and 25–75 percentiles according to their distribution. Then, qualitative variables were described using absolute and relative frequencies. To collect data, Research Electronic Data Capture (REDCap; Vanderbilt University, Nashville, TN, USA) was used, and IBM SPSS Statistics for Windows, Version 21.0 (IBM Corp., Armonk, NY, USA) was used for the analyses.^19^

## Results

### Sample Characterization and Clinical Features

A total of 12 ovarian SLCT patients were treated or referred to the HCB between October 2009 and August 2017. The median age was 31 (15–71) years old at diagnosis, while the median body mass index (BMI) was 26.7 (14.9–35.9) kg/m^2^. A total of 3 (25.0%) patients were asymptomatic; however, 8 (66.7%) patients reported abdominal pain, 5 (41.7%) presented with abdominal distention, 2 (16.7%) presented with signs of virilization, 2 (16.7%) presented with abnormal uterine bleeding during the menacme, 1 (8.3%) presented with dyspareunia, and 1 (8.3%) presented with weight loss. It is important to note that some patients presented > 1 symptom at admission to the HCB, as detailed in Table 1. The only sign of virilization reported in the present sample was hirsutism, with no cases of clitoromegaly, of voice alteration, or of weight gain. Amenorrhea occurred in 3 (25.0%) patients; however, this classification did not apply to 4 patients, because at the time of diagnosis 2 of them were pregnant, and 2 were already at menopause. Thus, regarding the hormonal profile, 10 (83.3%) patients were in the menacme, and 2 (16.7%) in the menopause. A total of 4 (33.3%) patients used hormonal contraceptives, and 1 (8.3%) had hormone replacement therapy. One (8.3%) patient was a smoker; however, there were no alcoholics in the sample. Two (16.7%) patients had a family history of gynecological cancer. Seven (58.3%) patients had physical exam abnormalities, which were: 5 (41.7%) with palpable abdominal mass, 1 (8.3%) with hirsutism, and 1 (8.3%) with acne.

**Table 1:** Clinical features of 12 patients diagnosed with ovarian Sertoli-Leydig cell tumors

N°	Age (years old)	Complaints at admission								Physical examination			
		Asymptomatic	Abdominal pain	Virilization	AUB	Abdominal distension	Dyspareunia	Amenorrhea	Weight loss	Normal	Palpable abdominal mass	Hirsutism	Acne
1	24	N	Y	N	Y	N	N	N	N	Y	N	N	N
2	17	N	Y	N	N	Y	N	Y	Y	Y	N	N	N
3	28	N	Y	N	N	N	Y	Y	N	Y	N	N	N
4	63	N	N	Y	N	N	N	N/A	N	Y	N	N	N
5	16	Y	N	N	N	N	N	N	N	Y	N	N	N
6	33	N	Y	N	N	N	N	N/A	N	Y	N	N	N
7	15	N	Y	N	N	Y	N	N	N	N	Y	N	N
8	15	N	Y	Y	N	Y	N	Y	N	N	Y	Y	Y
9	71	Y	N	N	N	N	N	N/A	N	N	Y	N	N
10	44	N	Y	N	N	Y	N	N	N	N	Y	N	N
11	34	N	Y	N	Y	Y	N	N	N	N	Y	N	N
12	40	Y	N	N	N	N	N	N/A	N	Y	N	N	N

Abbreviations: AUB, abnormal uterine bleeding; N, No; N/A, Not applicable; Y, Yes.

### Preoperative Tumor Markers

Preoperative tumor markers were measured in 6 patients (Table 2). The median dose of lactate dehydrogenase (LDH) was 504.5 U/L (138.0–569.0 U/L), alpha-fetoprotein (AFP) was 2.0 ng/ml (1.2–11.4 ng/ml), human chorionic gonadotropin (β-hCG) was 0.6 mUI/ml (0.0–2.4 mUI/ml), carcinoembryonic antigen (CEA) was 0.9 ng/ml (0.8–3.4 ng/ml), cancer antigen 125 (CA-125) was 26.0 U/ml (19.1–147.0 U/ml), total testosterone was 3.5 ng/ml (3.5–3.5 ng/ml) and dehydroepiandrosterone sulfate (DHEAS) was 224.0 μg/dl (224.0–224.0 μg/dl).

**Table 2:** Preoperative exams of 12 patients diagnosed with ovarian Sertoli-Leydig cell tumors

N°	Preoperative serum tumor markers							Preoperative imaging examination			
	LDH	AFP	β-hCG	CEA	CA-125	Total testosterone	DHEAS	Lesion size	Laterality	Morphology	Suspicious lymph nodes
	(U/L)	(ng/ml)	(mUI/ml)	(ng/ml)	(U/ml)	(ng/ml)	(µg/dl)	(cm)			
1	138.00	1.16	0.00	0.77		-	-	6.50	Left	Solid	No
2	-	-	0.00	1.00	42.00	-	-	-	-	-	-
3	-	-	-	-	-	-	-	-	-	-	-
4	-	-	-	-	-	-	-	-	-	-	-
5	-	-	-	-	-	-	-	-	-	-	-
6	-	-	-	-	-	-	-	11.70	Right	Solid-cystic	No
7	-	-	-	-	-	-	-	-	Left	-	-
8	465.00	11.38	0.00	0.79	26.01	3.55	224.00	20.00	Left	Solid-cystic	No
9	569.00	6.02	1.27	3.45	24.50	-	-	4.00	Right	Solid	No
10	-	2.05	2.39	2.15	19.10	-	-	13.00	Right	Cystic	No
11	544.00	1.61	2.39	0.93	147.00	-	-	24.00	Left	Cystic	No
12	-	-	-	-	-	-	-	23.10	Left	Cystic	-

Abbreviations: AFP, alpha-fetoprotein; β-hCG, human chorionic gonadotropin; CA-125, cancer antigen 125; CEA, carcinoembryonic antigen; DHEAS, dehydroepiandrosterone sulfate; LDH, lactate dehydrogenase.

### Preoperative Image Exams

Abdominal evaluation data were obtained from 8 patients (Table 2). This evaluation was performed by magnetic resonance imaging (MRI), computed tomography (CT) of the abdomen, transabdominal ultrasound and/or transvaginal ultrasound. In all of the patients with reviewed preoperative examinations, the lesions were unilateral, 3 (37.5%) to the right side, and 5 (62.5%) to the left side. Lesion morphology was described in 7 patients, and 3 (42.8%) had cystic lesions, 2 (28.6%) had solid lesions, and 2 (28.6%) had solid-cystic lesions. The median lesion size was 13 cm (4–24 cm). There were no cases of suspicious lymph nodes.

### Treatment

The initial treatment consisted of surgery for all patients. A total of 5 (41.7%) patients underwent primary surgery at the HCB, 5 (41.7%) patients were managed at another hospital and then were referred for follow-up at the HCB, and 2 (16.6%) patients were managed at another service and subsequently underwent surgical staging at the HCB. The time elapsed between the surgery in another service and the surgical staging at the HCB of these 2 patients was of 155 and 328 days. The surgical approach was laparotomic in 7 (70.0%) cases, and laparoscopic in 3 (30.0%) cases, with no conversions. It is important to mention that in 2 patients managed by surgery in another hospital, the surgical approach was not described in their medical records. Unilateral oophorectomy, unilateral salpingectomy, unilateral salpingo-oophorectomy, BSO, and total hysterectomy (TH) were some of the surgical procedures performed, which are described individually in Table 3. In addition, eventually, other surgical procedures were performed to complement staging, such as: omentectomy in 4 (33.3%) patients, pelvic or para-aortic lymphadenectomy in 3 (25.0%) patients, peritoneal biopsy in 2 (16.6%) patients and peritoneal washing in 6 (50.0%) patients. Two (16.6%) patients had intraoperative complications, which were hepatic cyst bleeding, controlled with electrocautery, and removal of the epiploic appendix with arterial bleeding. It is important to highlight that 2 (16.6%) patients were pregnant at the time of diagnosis. One of them was submitted to a right salpingo-oophorectomy during a cesarean section in another hospital, and then underwent surgical staging at the HCB. The other patient was submitted to excision of a left ovarian cyst during a cesarean section and then underwent surgical staging. Both surgeries were performed in another hospital. All of the surgical procedures performed are described in Table 3.

**Table 3:** Histopathological and treatment features of 12 patients diagnosed with ovarian Sertoli-Leydig cell tumors

N°		Surgery		Histopathology				
	Locality	Surgical approach	Surgery performed	Degree of differentiation	Tumor dimension (mm)	Characteristics	Mitotic rate (/10HPF)	Staging
1	HCB	-	Left oophorectomy, peritoneal washing	G2	5.50	-	18	IA
2	Other	Laparotomy	RSO, omentectomy	G2	-	Cystic with vegetations	-	IA
3	Other	Laparotomy	LSO	G2	-		-	IA
4	Other	Laparotomy	LSO	G2	4.00	Solid-cystic	-	IA
5	Other	Laparotomy	Right oophorectomy	G3	14.20	Solid-cystic	15	IA
6	Other/HCB	Laparoscopy	TH, BSO, omentectomy, peritoneal washing	G2	15.60	Solid-cystic	6	IA
7	Other/HCB	Laparotomy	LSO, para-aortic lymphadenectomy, peritoneal biopsy, peritoneal washing	G2	17.00	Solid-cystic	-	IA
8	HCB	Laparotomy	LSO, omentectomy, pelvic lymphadenectomy, peritoneal biopsy, peritoneal washing	G3	16.50	Solid-cystic	30	IA
9	HCB	Laparoscopy	BSO, peritoneal washing	G2	3.20	Solid	-	IA
10	HCB	Laparoscopy	RSO, left salpingectomy	G3	10.20	Cystic	-	IA
11	HCB	Laparotomy	LSO, cholecystectomy	G2	23.50	Cystic	-	IA
12	Other	-	TH, BSO, omentectomy, lymphadenectomy, appendectomy	G1	-	-	-	IA

Abbreviations: BSO, bilateral salpingo-oophorectomy; G1, well-differentiated; G2, moderately differentiated; G3, poorly differentiated; HCB, Hospital do Câncer de Barretos; HPF, high-power fields; LSO, left salpingo-oophorectomy; RSO, right salpingo-oophorectomy; TH, total hysterectomy.

### Histopathological Features

All of the tumors were confined to one ovary and were at stage IA (Table 3). Macroscopic analysis was available for 9 patients, of whom 5 (55.6%) had solid-cystic components, 2 (22.2%) had only cystic components, 1 (11.1%) had cystic components with vegetation, and 1 (11.1%) had only solid components. The median tumor size was 14.2 cm (3.2–23.5 cm), with 2 cases presenting < 5 cm, 6 cases > 10 cm, and only 1 case between 5 and 10 cm. One (8.3%) tumor was well-differentiated, 8 (66.7%) were moderately differentiated, and 3 (25.0%) were poorly differentiated. One case presented heterologous elements, such as atypical proliferative mucinous tumor (borderline), but no case in the present series showed a retiform pattern. The mitotic rate was measured in 4 patients, being 6/10 high-power fields (HPF), 15/10 HPF, 18/10 HPF, and 30/10 HPF. In the present sample, there were no cases of lymph node or of omental involvement, neither of peritoneal carcinomatosis. In addition, abdominal cytology was negative in 100% of the cases.

### Adjuvant Treatment and Follow-Up

Two (16.7%) patients received adjuvant treatment. One of them had a poorly differentiated tumor and underwent 3 cycles of paclitaxel and carboplatin every 21 days, and the other was strongly symptomatic at admission to the HCB and received 4 cycles of ifosfamide, cisplatin and etoposide every 21 days. Information about follow-up was available for all 12 patients in this series (Table 4). The length of follow-up varied between 1.6 and 65.0 months, with a median of 19.5 months. There were no cases of tumor recurrence. A total of 10 (83.4%) patients were alive and with no evidence of disease, and 1 (8.3%) was alive and was receiving adjuvant treatment. One (8.3%) patient presented mesenteric torsion 2 months after the surgical staging and underwent an exploratory laparotomy with segmental enterectomy and laterolateral anastomosis at the HCB. On the 3^rd^ postoperative day, this patient died after developing a septic and hemorrhagic shock. Thus, this death was considered related to the surgical staging, because there was no evidence of disease in the final pathology report.

## Discussion

In the present study, the median age at the time of diagnosis ranged from 15 to 71 years old, and abdominal pain was the most prevalent symptom in the present sample. A palpable abdominal mass was the predominant clinical sign in the physical exam. All of the tumors were at stage IA and were surgically treated. Regarding the histopathological features, most of the tumors were solid-cystic and moderately differentiated. Two patients received adjuvant chemotherapy; however, there were no cases of tumor recurrence, and one death related to surgical staging occurred.

**Table 4:** Follow-up of 12 patients diagnosed with ovarian Sertoli-Leydig cell tumors

N°	Adjuvant treatment	Recurrence	Length of follow-up (months)	Status
1	No	No	1.60	Alive, no evidence of disease
2	No	No	13.50	Alive, no evidence of disease
3	No	No	10.10	Alive, no evidence of disease
4	No	No	28.00	Alive, no evidence of disease
5	No	No	65.00	Alive, no evidence of disease
6	No	No	36.60	Alive, no evidence of disease
7	Ifosfamide + cisplatin + etoposide	No	12.50	Death related to surgical staging
8	No	No	31.50	Alive, no evidence of disease
9	No	No	31.70	Alive, no evidence of disease
10	Paclitaxel + carboplatin	No	3.40	Alive, but in treatment
11	No	No	12.30	Alive, no evidence of disease
12	No	No	25.60	Alive, no evidence of disease

Xiao et al^8^ divided the clinical manifestations found in their series of ovarian SLCTs into three categories: feminization, defeminization, and virilization. Manifestations of feminization included irregular vaginal bleeding, menorrhagia, or postmenopausal bleeding. Amenorrhea corresponded to a manifestation of defeminization, whereas facial pilification and clitoromegaly would be manifestations of virilization.^8^ In our study, the patients fit into all three categories described by this author: we had cases of feminization, exemplified by two patients with abnormal uterine bleeding; amenorrhea occurred in three patients, representing the manifestations of defeminization; hirsutism was the sign of virilization evidenced in the physical examination of one patient.

The age group most affected by this type of tumor corresponds to the 2^nd^ and 3^rd^ decades of life, predominantly, as evidenced in our study, whose median age at diagnosis was 31 years old.^4^ ^6^ ^11^ ^20^ In addition, the signs and symptoms are diverse and, associated with the low incidence of this type of neoplasia, the clinical diagnosis is also difficult. In our study, half of the patients had no hormonal manifestations, and the disease presented asymptomatic or only with abdominal pain, palpable mass or abdominal distension; in second place, androgenic manifestations appeared, such as amenorrhea, oligomenorrhea, hirsutism, voice alterations, laryngeal protuberance or clitoromegaly, and a minority of patients had estrogenic manifestations, such as abnormal uterine bleeding. Thus, when these manifestations appear in the clinical practice, ovarian SLCTs should be considered at least as a differential diagnosis, so that these tumors are not neglected and that the correct treatment can be implemented (Table 5).

**Table 5:** Review of published studies about ovarian Sertoli-Leydig cell tumors

Study	Median age (years old)	No hormonal manifestations (%)^a^	Androgenic manifestations (%)^b^	Estrogenic manifestations (%)^c^	Sample size
Roth et al^2^	24.5	15 (44.12)	15 (44.12)	4 (11.76)	34
Gui et al^4^	28	9 (22.50)	25 (62.50)	6 (15.00)	40
Nam et al^15^	31	7 (63.63)	3 (27.27)	1 (9.10)	11
Zhang et al^21^	-	3 (18.75)	7 (43.75)	6 (37.50)	16
Present case series	31	6 (50.00)	4 (33.30)	2 (16.70)	12

a asymptomatic or only abdominal pain, palpable mass or abdominal distension.

b amenorrhea, oligomenorrhea, hirsutism, voice alterations, laryngeal protuberance, clitoromegaly.

c abnormal uterine bleeding.

Alpha-fetoprotein consists of a glycoprotein detected at low levels in the blood of healthy adults. The liver and the yolk sac are its main sources. When present at serum levels above the standard value, it may be evidence of hepatocarcinoma or of germ cell tumors, such as the ovary and the testis. This glycoprotein is already a well-established tumor marker, being very useful in the diagnosis and in the follow-up of oncologic patients.^22^ In a study developed by Schneider et al,^13^ a high proportion of AFP-secreting ovarian SLCTs was observed. Sigismondi et al^14^ measured serum AFP values in 13 patients, and the tumor marker was elevated in 3 of them. In our study, the AFP dosage was available for five patients, and was elevated in one of them. These data suggest that ovarian SLCTs should be included in the clinical investigation of physicians as a differential diagnosis of AFP-secreting tumors, since when diagnosed in more advanced stages they are difficult to treat.

The treatment of ovarian SLCTs still remains controversial due to the paucity of cases described and to the limitation of the knowledge about the clinical behavior, the management, and the prognosis of this disease. Colombo et al^23^ suggested surgery as the standard primary treatment for this type of ovarian tumor. Because most SLCTs are unilateral and restricted to the ovary, fertility-preserving surgery with unilateral salpingo-oophorectomy and staging in patients who want to keep their fertility potential with no extraovarian disease are possible. The author also emphasizes the importance of endometrial evaluation to exclude concomitant endometrial neoplasia.

Colombo et al^23^ also suggested that for women in the postmenopausal period, with a more advanced stage of disease or with a bilateral tumor, hysterectomy and BSO should be performed, as well as a meticulous surgical staging. Besides that, exploration of the abdominal cavity, collection of fluid for cytological analysis, multiple peritoneal biopsies, omentectomy and dissection of pelvic and para-aortic lymph nodes need to be performed. Efforts should be expended for complete tumor cytoreduction. However, the role of surgical staging is controversial, considering the benefits of detailed surgical staging and the morbidity associated with high complexity procedures. Brown et al^16^ demonstrated that lymphadenectomy may be omitted from the surgical staging of patients with ovarian sex cord-stromal tumors, because lymph node metastases are rare and do not justify the potential surgical risk. In our sample, one patient underwent staging surgery with left salpingo-oophorectomy, para-aortic lymphadenectomy, peritoneal biopsy and peritoneal washing and developed mesenteric torsion 2 months later. This patient required further surgery with segmental enterectomy and died on the 3^rd^ postoperative day due to a septic and hemorrhagic shock. Thus, the type of surgical staging has to be discussed and individualized in order to reduce the morbidity and mortality associated with extensive surgical procedures.

Regarding adjuvant treatment, Colombo et al^23^ concluded that chemotherapy should be considered for poorly differentiated ovarian SLCTs, with heterologous elements or in advanced stages. In our study, adjuvant treatment was performed in two patients, in whom the tumors were poorly and moderately differentiated; however, none of them had heterologous elements in histopathology. Also, in the study by Schneider et al,^13^ out of 24 tumors at stage IA, including well-, moderately, and poorly differentiated, only 1 received adjuvant treatment, and none had recurrence. In our study, in our three patients with poorly differentiated tumors, only one received adjuvant treatment; however, all of them were at stage IA, and there was no tumor recurrence.

However, for the correct indication of adjuvant treatment, it is necessary to understand the prognostic factors of this disease. The low incidence of ovarian SLCTs makes it difficult to perform randomized studies evaluating the role of adjuvant treatment in more advanced tumors, as well as in patients at stage I with poor prognostic factors. In addition, the rarity of this type of tumor hinders the complete understanding of its prognostic factors.

Young et al^3^ discussed some prognostic factors for ovarian SLCTs. The findings of the authors show that the mitotic rate can predict a poor prognosis when > 15/10 HPF. In the present series, 3 patients had a mitotic rate ≥ 15/10 HPF; however, there was no evidence of recurrence throughout the whole length of the follow-up, whose median was of 19.5 months. It is also worth noting that in ovarian SLCTs, recurrences usually appear early, with 70% of the cases occurring in the 1^st^ year after treatment, and only 7% after 5 years.^1^

The degree of histological differentiation was also reported as a prognostic factor in ovarian SLCTs.^2^ ^3^ In a group of 207 cases, Young et al^3^ showed that the malignancy potential was 0%, 11%, and 59% for well-, moderately, and poorly differentiated tumors, respectively, and 19% for those with heterologous elements. Stage was another prognostic factor reported in the literature. In the MITO study, 70% of the patients with advanced stage had a disease-related death, whereas the 5-year survival rate was 92.3% for those at stage I.^14^ As mentioned previously, in the study by Roth et al,^2^ age and degree of histological differentiation were statistically significant as risk factors.

Our study is limited by the small sample number, considering the low incidence of this type of tumor, as well as by the retrospective characteristic, which implies a possible bias of data collection, since some information may not be contained in the medical records of the patients. However, our results are derived from a single institution, which is a reference in cancer treatment in Brazil and, thus, has standard procedures and routines in the management of the patients.

## Conclusion

In our series of 12 cases of ovarian SLCTs, abdominal pain was the most common clinical presentation and, in the physical examination, the presence of a palpable abdominal mass was the most frequent finding. All of the tumors were unilateral and there was no ultrasonographic pattern. Surgery was the treatment of choice for all patients, all of the cases were stage IA at the time of diagnosis, and most of the tumors were moderately differentiated. Two patients received adjuvant treatment and there were no recurrences. There was only one death, which was related to the surgical staging and not directly related to the ovarian neoplasia, since there was no evidence of residual disease in the final pathology. It is important to emphasize that new researches need to be developed to try to better understand the diagnosis, the staging and the prognosis of this rare disease and, thus, to try to better define therapeutic strategies.
